# Higher clozapine concentration-to-dose ratios at week 1 after initiation are associated with increased risk of clozapine-associated fever in Japanese patients with schizophrenia

**DOI:** 10.3389/fpsyt.2025.1591568

**Published:** 2025-05-30

**Authors:** Yuki Kikuchi, Hiroaki Tanifuji, Mutsumi Sakata, Kazuro Ikawa, Daisuke Kume, Naoki Horikawa, Sota Ueno, Yoshifumi Onuma, Masato Ishihara, Bunichiro Onodera, Takeshi Toraiwa, Hiroshi Komatsu, Toshihiro Sato, Masamitsu Maekawa, Nariyasu Mano, Hiroaki Tomita

**Affiliations:** ^1^ Department of Psychiatry, Graduate School of Medicine, Tohoku University, Sendai, Japan; ^2^ Department of Psychiatry, Kodama Hospital, Ishinomaki, Japan; ^3^ Department of Pharmacy, Kodama Hospital, Ishinomaki, Japan; ^4^ Nozoe Hills Hospital, Fukuoka, Japan; ^5^ Department of Clinical Pharmacotherapy, Hiroshima University, Hiroshima, Japan; ^6^ Department of Psychiatry, Tohoku University Hospital, Sendai, Japan; ^7^ Department of Pharmaceutical Sciences, Tohoku University Hospital, Sendai, Japan

**Keywords:** adverse effect, c-reactive protein, inflammation, pneumonia, myocarditis

## Abstract

**Introduction:**

The clinical use of clozapine in the treatment of schizophrenia is sometimes halted due to its inflammatory side effects. However, no method has been established for preemptively identifying patients who are at high risk for these events. Therefore, in this study, we assessed whether the blood clozapine concentration-to-dose ratio (C/D) on day 8 after initiation could predict the risk of clozapine-associated fever.

**Method:**

Patients whose blood levels of clozapine were measured on day 8 after initiation were retrospectively investigated. The C/D ratio on day 8 was compared between patients who had fever after day 8 and those who did not.

**Results:**

A total of 43 patients were included in the study. The range of C/D ratios on day 8 was from 0.35–4.0, with a median of 1.65. Patients with fever had significantly higher C/D ratios on day 8 than those without fever (2.33 vs. 1.47; P < 0.005).

**Conclusion:**

Measuring blood clozapine levels on day 8 may help to better estimate the risk of clozapine-associated fever in patients with schizophrenia.

## 1 Introduction

Clozapine is the most effective drug for treatment-resistant schizophrenia. However, its use is sometimes halted due to various adverse reactions, including inflammatory side effects during the early stages of titration. Clozapine-induced fever is a relatively common side effect. In some cases, it can progress to serious health conditions involving organ damage, such as pneumonia, myocarditis, nephritis, enteritis, pancreatitis, and skin rash (1). Relative fatality rates of pneumonia and myocarditis associated with clozapine have been reported to be higher than those due to agranulocytosis (2, 3). International guidelines to prevent the inflammatory adverse effects of clozapine have been established (4). Approximately 30% of Japanese patients developed a fever at the titration speed recommended by the protocol in Japanese package inserts (5). The Japanese package insert protocol states that clozapine should be started at 12.5 mg/day, increased by 25 mg/day every few days, and reach 200 mg/day on day 21. We have previously reported that slower clozapine titration prevents adverse reactions in Japanese patients (5–9). However, even with slower titration, approximately 10% of Japanese patients have been reported to develop inflammatory side effects (5, 8). No method has been established for pre-emptively identifying patients at high risk for this adverse reaction to clozapine. Monitoring C-reactive protein (CRP) levels weekly for four weeks after clozapine initiation is beneficial (4, 10). However, in some patients, CRP does not work as a marker to detect inflammation, as the increase in CRP levels can sometimes occur after the onset of inflammation (11). These adverse events often occur on days 10–20 after clozapine initiation. Therefore, identifying high-risk patients before this time window is necessary. In this study, we hypothesized that patients with inflammatory adverse events may have lower clozapine metabolic capacity. Clozapine concentration-to-dose (C/D) ratios can be measured on day 8, before the predicted onset of inflammatory adverse events and may help to predict the risk of such events. Therefore, we retrospectively investigated the relationship between the clozapine C/D ratio on day 8 after starting clozapine and the subsequent occurrence of clozapine-related fever.

## 2 Methods

### 2.1 Study participants

We retrospectively reviewed medical records from Nozoe Hills Hospital and Kodama Hospital. The inclusion criteria were as follows: 1) patients with treatment-resistant schizophrenia who received clozapine for the first time at Nozoe Hills Hospital between April 2020 and July 2024, and at Kodama Hospital between July 2009 and November 2024; 2) patients whose clozapine blood levels were measured on day 8 after initiating clozapine treatment.

In Japan, clozapine must be initiated upon hospitalization. The patients’ vital signs and their adherence to medication were monitored by nurses every day. When clozapine treatment is initiated, treatment-resistant schizophrenia must be diagnosed by a Clozaril Patient Monitoring Service (CPMS)-certified psychiatrist at a CPMS-certified hospital. The diagnosis of schizophrenia was based on the International Classification of Diseases, 10th revision. In Japan, clozapine is indicated for the following two types of treatment-resistant schizophrenia: (a) patients who do not achieve a Global Assessment of Functioning score ≥41 after at least 4 weeks of treatment with two or more antipsychotics (chlorpromazine equivalent ≥600 mg); or (b) patients who do not achieve sufficient therapeutic effects from monotherapy with two or more atypical antipsychotics because the dose could not be increased to a sufficient level due to the development of extrapyramidal symptoms. No patients presented with catatonic symptoms such as akinesia, stupor, or muscle rigidity before or after clozapine treatment, nor were any patients diagnosed with neuroleptic malignant syndrome. Fever was defined as an armpit temperature of ≥37.5°C within 6 weeks of clozapine treatment initiation. One patient from Nozoe Hills Hospital was excluded from the cohort, as the patient developed a fever before day 8 after starting clozapine. A total of 43 patients (27 from Nozoe Hills Hospital and 16 from Kodama Hospital) were included in the study. The mean age (standard deviation) of the 43 patients was 33.9 (14.9) years, and 19 (44%) of the participants were male (**Table 1**).

**Table 1:** Patient demographics and C/D ratios on day 8.

	All	Nozoe Hills Hospital	Kodama Hospital
Number of patients, n	43	27	16
Male, n (%)	19 (44)	12 (44)	7 (44)
Age, year, mean (SD)	33.9 (14.9)	31.1 (16.1)	38.5 (11.4)
BMI, kg/m^2^, mean (SD)	24.5 (5.9)	23.4 (5.2)	26.3 (6.7)
Concomitant use of valproate, n (%)	8 (19)	7 (26)	1 (6.3)
Smoking, n (%)	7 (16)	4 (15)	3 (19)
Fever, n (%)	9 (21)	5 (19)	4 (25)
Fever onset, day, median (IQR)	16 (12-17)	12 (11–14)	17 (16.75-18)
Mean clozapine dose on days 3-7, mg, median (IQR)	40 (25–40)	40 (25–40)	25 (25–25)
C/D ratio on day 8, median (min, IQR, max)	1.65 (0.35, 0.99–2.26, 4.0)	1.46 (0.35, 0.98–1.91, 3.3)	2.13 (0.42, 1.37–2.13, 4.0)

BMI, body mass index; C/D ratio, concentration-to-dose ratio; IQR, interquartile range; SD, standard deviation; min, minimum; max; maximum.

- Smoking is prohibited at Nozoe Hills Hospital, and only three patients at Kodama Hospital were allowed to smoke during clozapine titration.

- The reasons why valproic acid is widely used in Japan for patients with treatment-resistant schizophrenia have been discussed in detail in a previous study (7).

As this retrospective study used anonymized data, an opt-out form was displayed on the hospital’s bulletin board before collecting the data, and individuals who did not express the intent for exclusion were included in the study. All procedures complied with the ethical standards of relevant national and institutional committees on human experimentation and with the 1975 Declaration of Helsinki, as revised in 2008. The research conducted at Nozoe Hills Hospital was approved by the Ethics Committee of Nozoe Hills Hospital (Approval ID: 2024-6). Similarly, the research conducted at Kodama Hospital was approved by the Ethics Committee of the Tohoku University Graduate School of Medicine (Approval ID: 2023-1-68).

### 2.2 Measurement of clozapine blood levels at Nozoe hills hospital

In most patients, blood was collected approximately 12 h after the last clozapine dose. The plasma concentrations of clozapine and norclozapine were measured using high–performance liquid chromatography at the Department of Clinical Pharmacotherapy, Hiroshima University, Hiroshima, Japan, as reported previously (12).

### 2.3 Measurement of clozapine blood levels at Kodama hospital

All patients were administered clozapine once daily in the evening. Blood clozapine levels were measured at trough time points on day 8 (blood was collected at approximately 3 pm). The measurement of blood concentrations of clozapine and norclozapine was outsourced to the LSI Medience Corporation (Tokyo, Japan) and performed using liquid chromatography-mass spectrometry. It took more than 4 days for the blood concentrations to be determined at both hospitals; therefore, the test results could not be used to adjust the clozapine dose immediately.

### 2.4 Assessment of C/D ratio

The average clozapine dose within 5 days before clozapine blood level measurements was used to calculate the C/D ratios. This is because the clozapine dose changed during titration, and the steady-state blood concentration measurements were not available. The C/D ratio on day 8 was compared between patients who had fever after day 8 and those who did not have fever.

### 2.5 Statistical analysis

Statistical analyses were performed using EZR software (Jichi Medical University, Saitama, Japan). The C/D ratios and clozapine/norclozapine ratio between the groups were tested using the Mann–Whitney *U* test. The significance level was set at a *P* value of < 0.05.

## 3 Results

**Figure 1:**
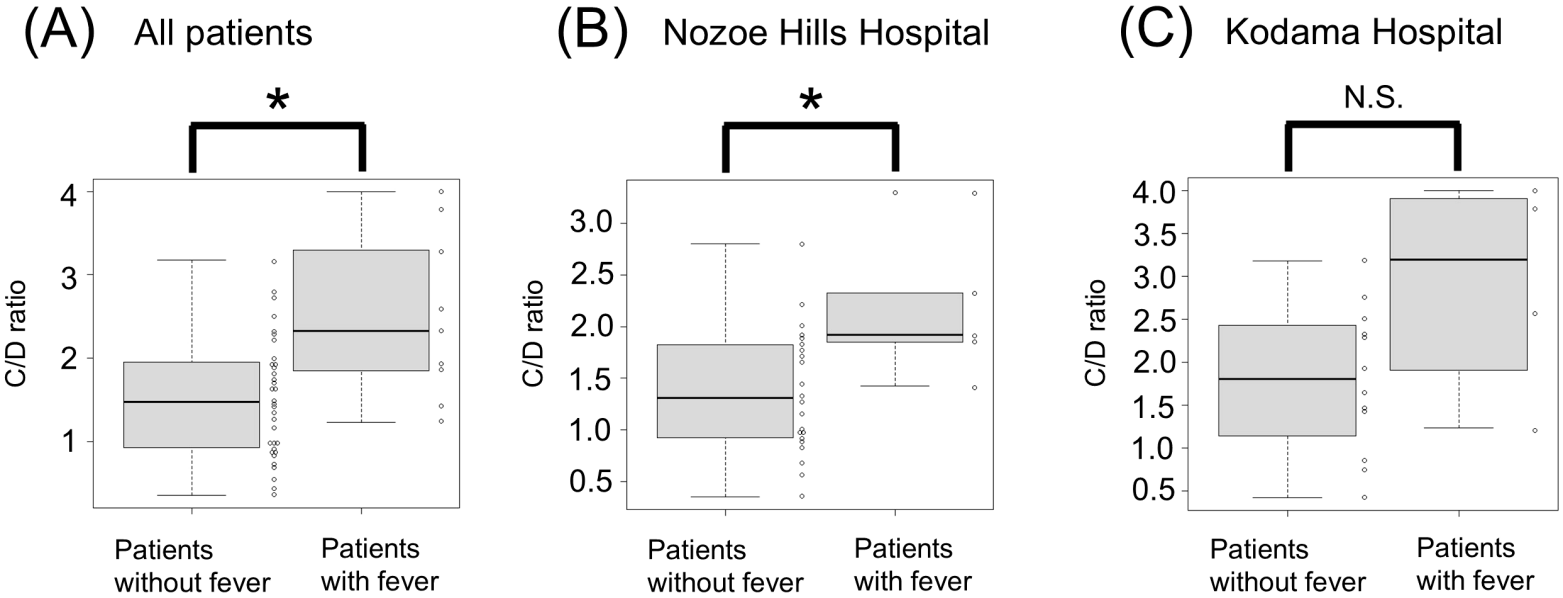
Comparison of C/D ratios between patients with and without fever. **(A)** All patients. **(B)** Patients in Nozoe Hills Hospital. **(C)** Patients in Kodama Hospital. Significant differences are indicated by asterisks, according to the Mann–Whitney U test. C/D ratio, concentration-to-dose ratio; N.S., not significant.

**Figure 2:**
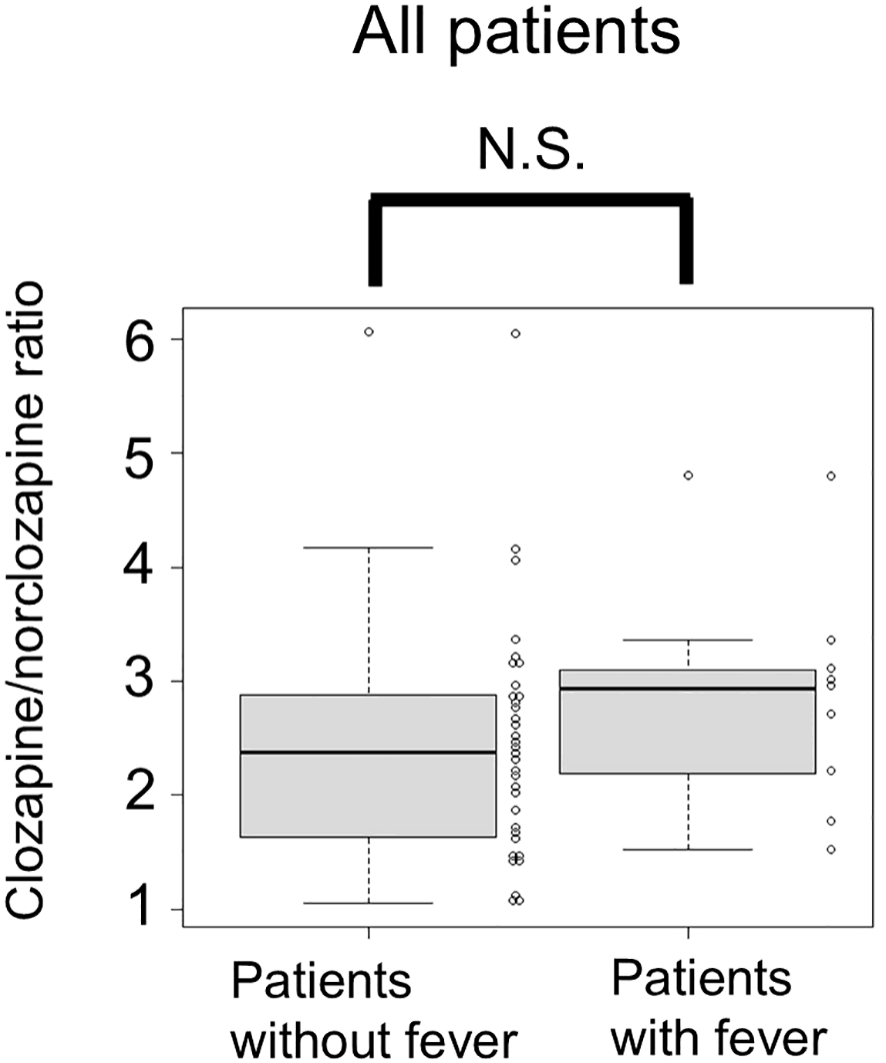
Comparison of clozapine/norclozapine ratios on day 8 between patients with and without fever. Among patients who did not develop fever, the blood concentration of norclozapine was below the limit of quantification in six patients. In these patients, the limit of quantification (10 ng/ml) was used in the calculation of the clozapine/norclozapine ratio. N.S., not significant.

**Table 1** shows demographic data for all patients. Of the total study participants (n = 43), nine patients (21%) experienced fever after the eighth day of starting clozapine. **Table 2** provides details on the nine patients who developed fever. The course of five patients with fever at Nozoe Hospital is described in detail in a previous study (12). The median (interquartile range [IQR]) of the average clozapine dose for the 5 days prior to the day of clozapine blood concentration measurement (day 8) was 40 (25–40) mg/day. C/D ratios on day 8 for individual patients ranged from 0.35 to 4.0, with a median (IQR) of 1.65 (0.99–2.26) (**Table 1**). The median C/D ratio of patients with fever was 2.33 (IQR 1.85–3.30), while the median C/D ratio of patients without fever was 1.47 (IQR 0.94–1.94) (**Figure 1A**). The C/D ratio was significantly higher in patients with fever than in those without fever (median C/D ratio 2.33 vs. 1.47; P < 0.05). As a sensitivity analysis, the same analysis was performed separately for each hospital. In Nozoe Hills Hospital, the median C/D ratio was significantly higher in patients with fever than that in those without (**Figure 1B**; median C/D ratio 1.92 vs. 1.31; P < 0.05). In Kodama Hospital, the median C/D ratio was higher in patients with fever than in those without fever, but this was not statistically significant (**Figure 1C**; median C/D ratio 3.19 vs. 1.80; P = 0.133). There was no significant difference in the clozapine/norclozapine ratio on day 8 between patients who developed fever and those who did not (median clozapine/norclozapine ratio (IQR): 2.94 (2.19–3.10) vs. 2.38 (1.65–2.87); P = 0.20) (**Figure 2**).

**Table 2:** Demographic data of nine patients with fever.

Patient	Hospital	Sex	Age	BMI (kg/m^2^)	Smoking^a^	Concomitant medications at start of clozapine^b^	C/D ratio on day 8	Daily dose of clozapine on day 8 (mg)	Fever onset (day)	Clozapine dosage on the day of fever onset (mg)	Fever duration (days)	Clinical diagnosis	Clozapine discontinuation
1	Nozoe	F	60s	17.4	–	Trihexyphenidyl Nitrazepam	1.85	50	11	75	5	Clozapine-induced fever	No
2	Nozoe	F	10s	20.3	–	Biperiden Nitrazepam	3.3	50	10	75	4	Clozapine-induced fever	No
3	Nozoe	F	50s	21.4	+	Estazolam	1.43	50	14	100	4	Pneumonia	Yes
4	Nozoe	M	30s	24.9	–	Biperiden Nitrazepam	2.33	50	12	100	2	Clozapine-induced fever	No
5	Nozoe	F	10s	25	–	Nitrazepam	1.92	50	20	50	1	Clozapine-induced fever	No
6	Kodama	F	20s	24.2	–	Olanzapine Brexpiprazole Nitrazepam	4.0	25	21	75	3	Clozapine-induced fever	No
7	Kodama	F	20s	44.2	–	Quetiapine Blonanserin Lamotrigine Venlafaxine Triazolam	2.58	25	16	100	1	Clozapine-induced fever	No
8	Kodama	F	20s	27.9	–	Asenapine Olanzapine Diazepam Lemborexant	3.8	25	17	75	2	Clozapine-induced fever	No
9	Kodama	M	40s	20.0	–	Risperidone Blonanserin Lithium Biperiden Lemborexant	1.23	25	17	75	4	Clozapine-induced fever	No

^a^Smoking status at Nozoe Hills Hospital refers to smoking prior to admission, since smoking was not allowed in the hospital.

^b^Clozapine and these antipsychotic medications were cross-titrated.

BMI, body mass index.

## 4 Discussion

This study revealed that the C/D ratios on day 8 after clozapine initiation were significantly higher in patients with fever than in those without fever. Therefore, this study suggests that the metabolic capacity of patients may be linked to the development of clozapine-associated fever. A Korean study has previously reported that a higher C/D ratio is a risk factor for the development of clozapine-associated inflammatory reactions (13), which is consistent with our findings. There was an overlap in C/D ratios between patients with and without fever. However, no patient with C/D ratios <1.23 developed a fever, and all three patients with C/D ratios >3.18 developed a fever.

In most patients, C/D ratios on day 8 can be measured before the predicted onset of inflammatory adverse events. In Japan, monthly blood concentration measurements of clozapine are covered by insurance; therefore, it is feasible to measure on day 8 after starting clozapine. Measuring blood clozapine levels on day 8 can help to predict the risk of such events. Based on the results of this study, we suggest that patients with a C/D ratio of 3–4 on day 8 have a significantly high risk of developing inflammatory adverse events. Slowing the titration speed after day 8 in patients with a high C/D ratio may be effective in reducing the risk of these events. However, even in this study, it took time to determine the blood concentrations of clozapine, and it was not possible to adjust the dose immediately. To apply the results of this study to clinical practice, it is necessary to establish a laboratory testing infrastructure that can assess and use blood clozapine concentrations immediately. Measuring blood concentrations inside the facility rather than outsourcing may be effective in shortening turnaround time and helping clinical decisions (14).

Previous studies have reported that approximately 10% of Japanese patients with schizophrenia treated with clozapine develop inflammatory adverse events, even with slow titration (5, 8). The present study suggests that patients at high risk of such events may have a high C/D ratio. In this cohort, the range of C/D ratios was 0.35−4, implying there is a difference of approximately 10-fold in individual metabolic capacity. Therefore, a fixed titration rate, such as the protocol in the package insert, is inappropriate. Instead, a tailored protocol needs to be considered for each individual.

Considering the careful monitoring required during clozapine titration, collaboration with clinical pharmacists can help to improve the management of patients taking clozapine (15). At Kodama Hospital, clinical pharmacists make rounds in the wards, ask patients about side effects, and work with psychiatrists to check blood test results (such as clozapine blood levels and CRP levels). They also discuss the clozapine titration speed.

Some patients with clozapine-associated drug reaction with eosinophilia and systemic symptoms (DRESS) syndrome are hypersensitive to even small doses of clozapine (1). In such cases, their C/D ratio may not be associated with the risk of inflammatory adverse events.

In the present study, no association between the clozapine/norclozapine ratio and subsequent fever was found. International guidelines have established the C/D ratio as the most reliable indicator of an individual’s clozapine metabolic capacity (4). For example, the C/D ratio is significantly higher in women than in men, in Asians than in Caucasians, and in non-smokers than in smokers, reflecting the lower clozapine metabolic capacity in these groups (4). However, the clozapine/norclozapine ratio has not been established as an indicator reflecting individual clozapine metabolic capacity (16). For example, sex and smoking have been repeatedly shown to have no significant effect on the clozapine/norclozapine ratio (16, 17). Multiple studies emphasize that the clozapine/norclozapine ratio should not be interpreted as specific information regarding CYP 1A2 activity (16, 18, 19). This is because multiple factors, such as multiple CYP types and the P-glycoprotein transporter, may be involved in the observed ratios (19). Furthermore, there are no studies showing longitudinal changes in the clozapine/norclozapine ratio within individuals (16). Therefore, the lack of an association between the clozapine/norclozapine ratio and subsequent fever in this study does not compromise the main findings of this study.

This study had several limitations. First, the number of cases in this study is small. At Kodama Hospital, the difference in the C/D ratio between patients with fever and those without was not statistically significant. The number of participants at Kodama Hospital (N = 16) was smaller than that at Nozoe Hills Hospital (N = 27), resulting in insufficient statistical power. Further research with larger sample sizes is required to confirm the findings of this study. Second, an average dose over the 5 days prior to blood concentration measurement was used to calculate the C/D ratio, as it is difficult to use steady-state concentrations during titration. Third, the study focused on Japanese patients, which lacked generalizability to other populations. Finally, this study does not clarify how individual differences or changes in metabolism affect the clozapine C/D ratio on day 8. Asymptomatic inflammation may increase the C/D ratio on day 8 before the onset of clinical symptoms of inflammation, but the underlying mechanism is also unclear in this study. Future studies should investigate the mechanism by which changes in metabolic pathways increase the C/D ratio on day 8 and the causal relationship between the increase in the C/D ratio on day 8 and fever (i.e., whether the increase in the clozapine blood level causes fever, whether fever causes an increase in clozapine blood level, or both).

In conclusion, measuring blood clozapine levels on day 8 may help to better estimate the risk of inflammatory adverse events caused by clozapine. Therefore, we recommend monitoring day 8 blood clozapine levels in patients with schizophrenia as part of clinical practice.

## Data Availability

The datasets presented in this article are not readily available because the data are not publicly available because they contain information that can compromise the privacy of the research participants. Requests to access the datasets should be directed to Yuki Kikuchi, ykikuchi@sand.ocn.ne.jp.
